# Mummies tell stories: CT investigation of eight members of the Kėdainiai elite (Grand Duchy of Lithuania, 17th-18th centuries AD)

**DOI:** 10.3389/fmed.2025.1532751

**Published:** 2025-04-29

**Authors:** Dario Piombino-Mascali, Rokas Girčius, Algirdas Tamošiūnas, Rimantas Jankauskas, Rūta Brindzaitė, Justina Kozakaitė

**Affiliations:** ^1^Cranfield Forensic Institute, Cranfield University, Cranfield, United Kingdom; ^2^Santaros Klinikos Vilnius University Hospital, Vilnius, Lithuania; ^3^Faculty of Medicine, Vilnius University, Vilnius, Lithuania; ^4^Faculty of History, Vilnius University, Vilnius, Lithuania

**Keywords:** bioarchaeology, embalming, mummification, paleopathology, paleoradiology

## Abstract

The objective of this study is to evaluate the mummified remains of eight high-ranking people buried in two crypts of the Evangelical Reformed Church at Kėdainiai, Lithuania. The evaluation criteria include biological or cultural indicators, the assessment of pathological conditions and their possible etiology, and the preservation status of these remains. The eight individuals were recovered during a project aimed at exploring the tombs of potential members of the Radziwiłł family, a powerful dynasty of the former Grand Duchy of Lithuania and the Crown of the Kingdom of Poland (1569–1795). However, the remains could also belong to other affluent citizens of Kėdainiai who were buried in the same church between the 17th and 18th centuries. The deceased were investigated using classical anthropological methods and computed tomography, which allowed for a more nuanced vision of both individual social status and bio-histories for this assemblage. The results identify one case of post-mortem manipulation, evidence of significant pathological changes, including degenerative joint disease, lung and arterial calcifications, and neoplasias that would not have been visible without a paleoradiological approach. The historical context, as well as comparative clinical cases, helped narrow down the diagnoses proposed for the lesions concerned, and will be crucial to address additional histological or biomolecular research, should this be carried out in the future. Additionally, the study highlights the need for regular monitoring of the remains, particularly given the evident decay observed over the past four decades. This adds to the body of research suggesting that the more frequent inspection of individuals in which socioeconomic status can be assumed through mortuary context is warranted. In sum, this investigation shows that paleopathology, coupled with paleoradiology, provides a more permanent data set that enhances the interpretation of pathological conditions in preserved bodies, especially when they are in physical danger due to environmental or political changes.

## Introduction

Ancient and historic mummified remains are a valuable source of bioarchaeological and biomedical information, offering insights into individual behavior, disease, and past funerary practices (1, 2). While such remains are found in various cultures worldwide, they are particularly common in religious buildings like Christian churches, which often contain subterranean chambers for the burial of distinguished members of society (3).

**Figure 1:**
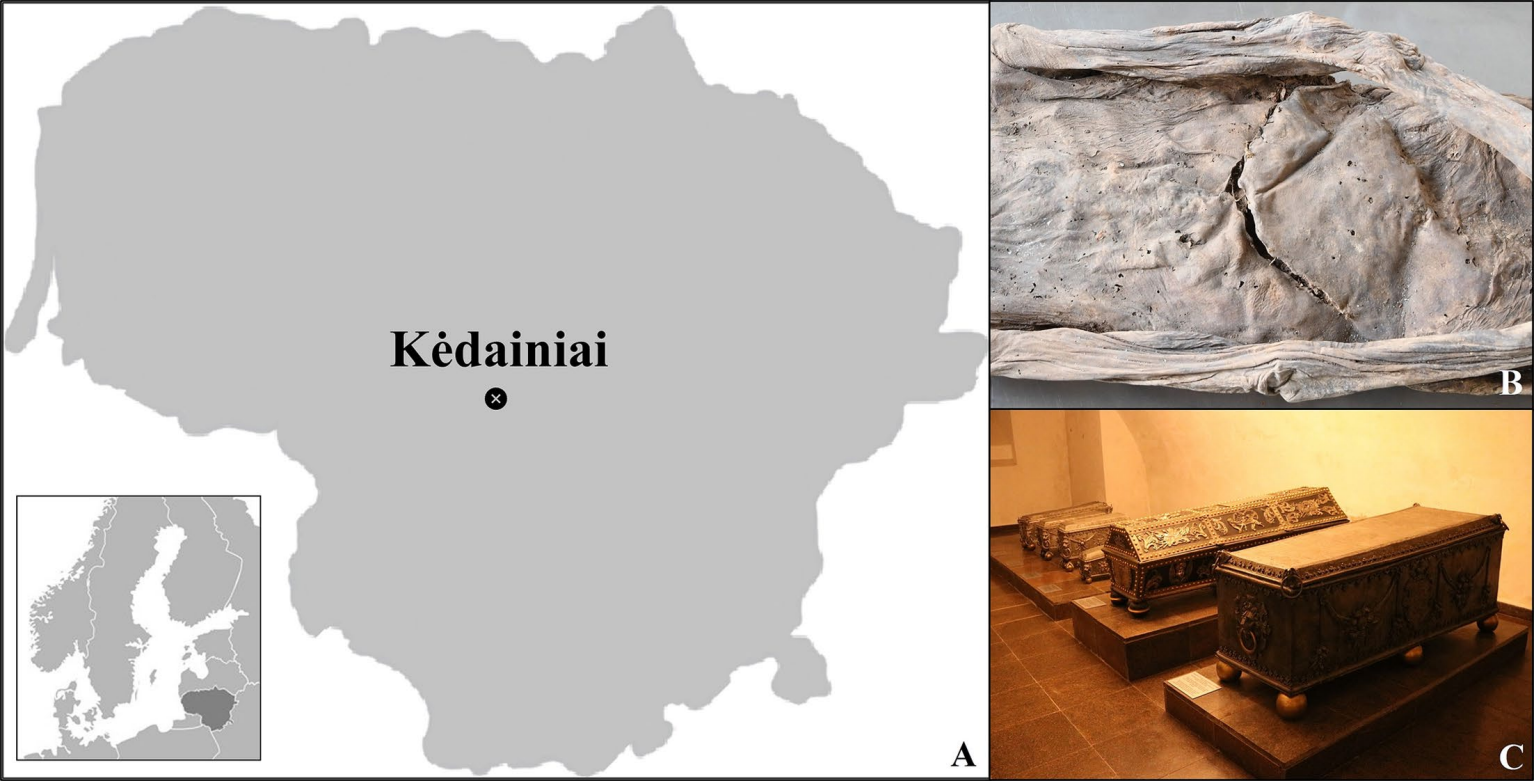
**(A)** Map of Lithuania showing the location of Kėdainiai; **(B)** The visible abdominal incision of individual #0; **(C)** The coffined remains exhumed during the onsite investigation.

The eight mummified individuals discussed in this paper, dating to the 17th and 18th centuries AD, were recovered from a crypt in the Evangelical Reformed Church of Kėdainiai, a historic town in central Lithuania (geographical coordinates: 55°17′N 23°58′E) (Figures 1A–C). This chamber was used over generations to bury members of the Radziwiłł family, a powerful dynasty of the former Grand Duchy of Lithuania and the Crown of the Kingdom of Poland (1569-1795). The church also contained another crypt, used for other dignitaries not related to the Radziwiłł family but living in the same context. Both rooms had been disturbed multiple times, particularly after WWII, when the building was converted into a sports hall, leading to potential mixing and desecration of the remains (4).

In July 2019, members of the Kėdainiai Regional Museum and the Faculty of History of Vilnius University began a project to inspect the remains from both crypts, with the goal of identifying the deceased and reinterring them with new coffins (Figure 1C). During this process, 37 bodies and body parts were recovered, including 29 mummified or partly mummified individuals and eight skeletonized ones. Of these, 18 were identified as males or probable males, 13 as females or probable females, and six had undetermined sex. Age estimates were made for 25 of the individuals, while the remaining mummified cases were categorized simply as “adults.” Additionally, there were 73 commingled skeletonized individuals. Previous inspections of these remains were conducted in 1986-1987, providing a reference point for evaluating their preservation status.

The primary aim of this research is to assess the health, social status, and cultural practices of the individuals buried in the crypts. The research questions guiding this study include: What can these remains reveal about the individuals’ health, social rank, and diseases? How do these findings fit within the broader historical, social, and biological context of the time? The specific objectives of this sub-project are to evaluate the social status of the deceased based on biological and cultural indicators, identify pathological conditions and their possible causes, and assess the preservation status of the remains for documentation purposes.

Paleoradiological imaging was used on the eight best-preserved mummified individuals to confirm their identity and examine the bone and soft tissue abnormalities observed. These activities were conducted with great care to avoid causing visible damage to the corpses, respecting ethical standards (5). This study offers new insights into the lives of these individuals, shedding light on their health, social roles, and the funerary practices of 17th- and 18th-century Lithuania.

## Materials and methods

Upon visual inspection, the eight individuals selected for this study showed no evidence of anthropogenic manipulation, indicating a process of spontaneous mummification, except for one case that revealed an abdominal incision (Figure 1B) (1). They were studied using standard bioanthropological methods, including sexual assessment based on cranial and pelvic features, inspection of the external genitalia, and age-at-death estimation using indicators such as changes in the sternal rib ends, and dental and long bone development (6–8). This assessment was not possible for all cases, as some of the anthroposcopic areas (e.g., the skull, the pelvis) were either missing or covered by desiccated soft tissue (9, 10). The remains were also examined for pathological changes visible on the skeletonized parts (11–13).

For this assemblage, computed tomography (CT) was chosen as the method of analysis due to its non-invasive nature and effectiveness in mummy research (14). The scanning was conducted at Santaros Klinikos Vilnius University Hospital using a 64-slice LightSpeed VCT scanner (GE Medical Systems), with a slice thickness of 0.6 mm at 120 kVp. Measurements were taken to ensure the safety of both the environment and the research team (15). The CT data were visualized, measured, and rendered using RadiAnt DICOM Viewer software (Medixant). Pathological alterations observed in the soft and hard tissues were described using a modified version of the standardized Istanbul protocol, which categorized signs as inconsistent, consistent, highly consistent, typical, or diagnostic of certain conditions (16).

Since the mummified remains were not in a standard anatomical position, it was not possible to depict them in a conventional plane system. Therefore, all CT images were created using multi-planar reconstructions, with planes shifted from multiple angles to provide the best possible view while sacrificing traditional plane orientation. In addition to pathological examination (1), stature estimation was performed by measuring the femur using the methodology devised by Trotter and Gleser (17) for European-ancestry populations. Biological samples from both soft and hard tissues, taken from areas affected by post-depositional changes, will be investigated in future studies.

## Results

### Biological features and preservation status

The selected sample consists of four males and four females (Table 1). These individuals are generally categorized as adults (~20–50 years), with the exception of one young adult (~20–35) and one middle adult (~35–50). The assemblage includes seven spontaneously mummified individuals and one anthropogenically mummified individual (#0), which, as previously mentioned, showed evidence of evisceration and embalming, including vegetal stuffing of the abdomen.

**Table 1:** Bioanthropological and paleopathological features of the sample concerned.

Individual	Sex	Age	Preservation	Right femoral length	Stature	Main pathological condition
#0	M	Middle adult (~35–50 years)	Anthropogenic	46.8 cm	172.8 cm (*σ* = ± 3.27 cm)	Degenerative, neoplastic (benign)
#1	M	Adult (~20–50 years)	Spontaneous, acephalous	47.0 cm	173.3 cm (*σ* = ± 3.27 cm)	Degenerative, genetic, respiratory
#2	F	Young adult (~20–35 years)	Spontaneous, partly skeletonized	44.4 cm	163.8 cm (*σ* = ± 3.72 cm)	Degenerative, neoplastic (benign)
#3	F	Adult (~20–50 years)	Spontaneous, acephalous	43.3 cm	161.1 cm (*σ* = ± 3.72 cm)	Degenerative, neoplastic (benign)
#4	F	Adult (~20–50 years)	Spontaneous, partly skeletonized	41.2 cm	155.9 cm (*σ* = ± 3.72 cm)	Cardiovascular, degenerative, genetic, neoplastic (benign)
#8	M	Adult (~20–50 years)	Spontaneous, acephalous, partly skeletonized	47.4 cm	174.2 cm (*σ* = ± 3.27 cm)	Cardiovascular, degenerative, neoplastic (benign), respiratory
#14	F	Adult (~20–50 years)	Spontaneous, partly skeletonized	39.9 cm	152.7 cm (*σ* = ± 3.72 cm)	Respiratory
#15	M	Adult (~20–50 years)	Spontaneous, acephalous	47.1 cm	173.5 cm (*σ* = ± 3.27 cm)	Respiratory

Most of the bodies involved soft tissue mummification, with exceptions: individual #2, whose upper body was skeletonized; individual #4, whose skull and lower legs were skeletonized; individual #8, whose right lower leg was skeletonized and the left was missing; and individual #14, whose skull, upper arms, and lower legs were skeletonized, with the left forearm skeletonized and the right missing. Four individuals (#1, #3, #8, and #15) lacked skulls, which may still be among the commingled remains. Based on body dimensions and skin folds, individual #4 appeared to be obese.

Lastly, the calculated height of the male individuals was notably higher compared to other contemporary Lithuanian samples (*M* = 172.8–174.2 cm; *F* = 152.7–163.8 cm) (18) (Table 1).

### Degenerative joint disease

Degenerative changes in the articular cartilages and various joint elements were observed in the assemblage, manifesting as bone formation or destruction. When synovial joints were affected, these changes reflected the onset of osteoarthritis (13, 19). Eburnation, which is pathognomonic, could not be identified in the CT scans and was therefore excluded as a diagnostic criterion. Joint space narrowing was also not considered, as it could be due to post-depositional alterations (20).

**Figure 2:**
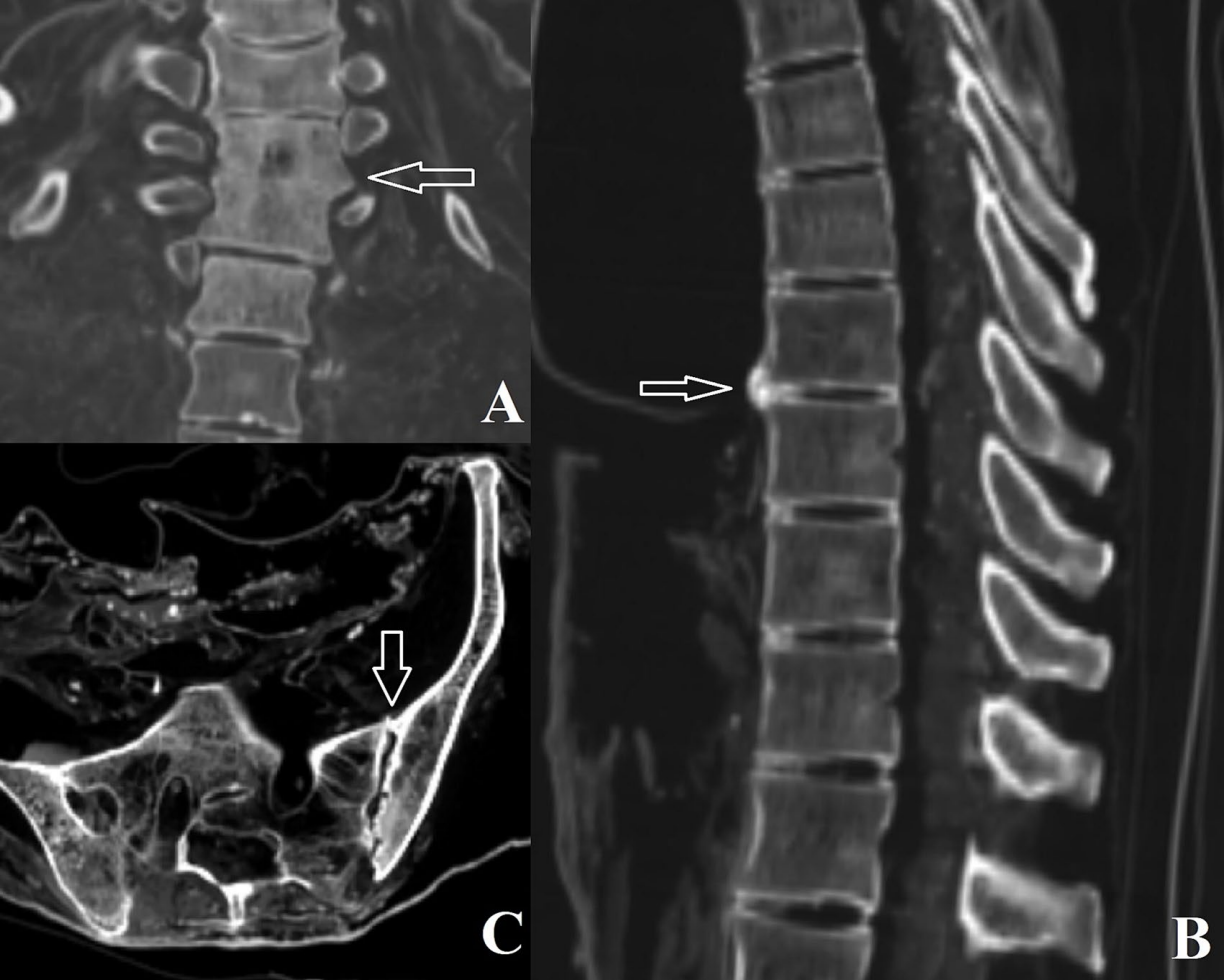
**(A)** Fusion of thoracic vertebrae 2 and 3 in individual #0; **(B)** Degenerative joint disease affecting thoracic vertebrae 8 and 9 in individual #3; **(C)** Left sacroiliac changes observed in individual #4.

Individual #0 showed mild degeneration of the cervical spine, moderate changes in the thoracic spine, and lateral fusion on both sides of the 2nd-3rd thoracic vertebrae (Figure 2A). Five Schmorl’s nodes were identified in the thoracic vertebrae, and two in the lumbar vertebrae. Individual #1 exhibited probable sacroiliitis, indicated by sclerosis of the subchondral bone. Individual #2 presented with a cyst at the base of the third right metatarsal. The third tarsal-metatarsal joint on the medial side was uneven, likely due to bone erosion, with a sclerotic margin at the lesion. This was associated with a subchondral degenerative bone cyst in the lateral cuneiform on the opposite side. These changes likely suggest post-traumatic osteoarthritis or joint-related problems (such as a ganglion). In individual #3, the thoracic segment showed mild degeneration, except at the 8th and 9th vertebrae, where a marginal right osteophyte was present (Figure 2B). Her left knee displayed mild sclerosis on the right side, possibly due to mild degeneration.

Individual #4 revealed cervical, thoracic, and lumbar changes in the form of osteophytes and lipping, as well as left sacroiliac and right acromioclavicular subchondral cysts and surface sclerosis (Figure 2C). Individual #8 showed spinal changes (cervical, thoracic, and lumbar), with apophyseal and intervertebral changes, as well as osteoarthritis in the right acromioclavicular joint. The left acromioclavicular joint space was wider, potentially indicating subluxation with post-traumatic articular surface changes or simple degeneration. In the latter case, the space widening may be post-mortem.

### Neoplasias

**Figure 3:**
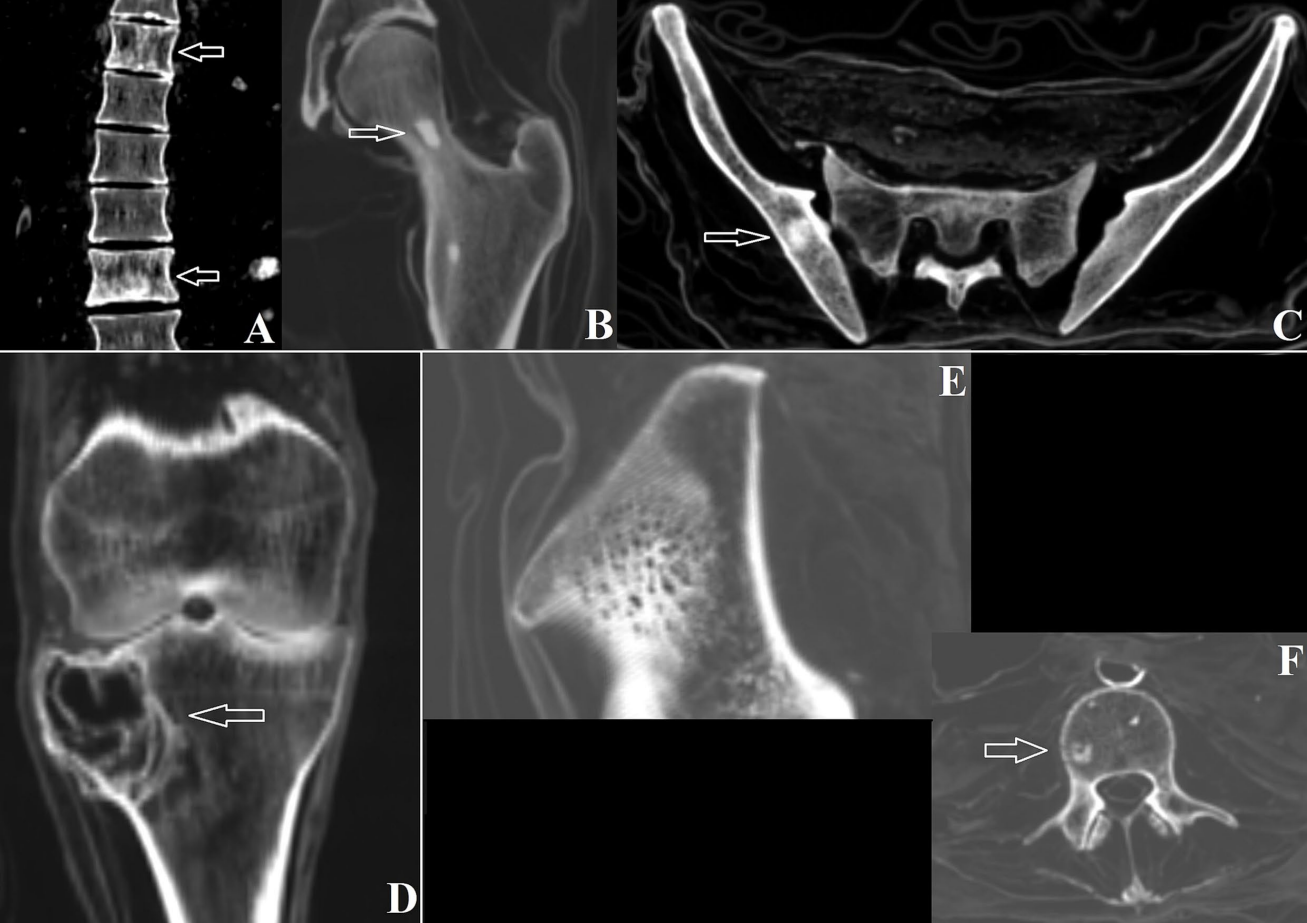
**(A)** Vertebral hemangiomas on thoracic vertebrae 6 and 10 in individual #0; **(B)** Bone island on the left femoral neck of individual #0; **(C)** Right bone island on the pelvic bone of individual #0; **(D)** Giant cell tumor in the right tibia of individual #3; **(E)** Intraosseous hemangioma in the right pelvis of individual #8; **(F)** Bone island on lumbar vertebra 3 of individual #8.

Tumors are groups of abnormal cells that form growths, which can be either benign or malignant (21). Evidence of tumor formation was found in some of the mummies. On the axial plane, individual #0 revealed “polka dot” lesions on the 6th and 10th thoracic vertebral bodies, while a sagittal view showed a vertically striated appearance due to thickening of the bony trabeculae. No cortical break or surrounding soft tissue mass was observed. These findings are strongly associated with vertebral hemangioma, a benign tumor formed by vascular tissue that most commonly affects the thoracic spine when present on bone (Figure 3A) (22).

This individual also exhibited multiple small, round-to-oval foci of homogeneously dense bone within the medullary space, with no cortical destruction or periosteal reaction. These are typical of bone islands (Figures 3B,C), which are lesions of mature cortical bone within the spongiosa, developmental or congenital in origin. Specifically, bone islands were seen on the right distal radius, scaphoid, ulna, right pelvis (12 mm), right distal tibia, right calcaneus, cuboid, and lateral cuneiform. The left femoral neck (10 mm), distal tibia, distal fibula, navicular, cuboid, and first metatarsal were also affected (23).

Individual #2 also revealed a sclerotic lesion on the body of the 12th thoracic vertebra (8 mm), demonstrating another bone island. Individual #3 showed an eccentric lesion with a circumscribed border, resulting in a small lateral cortical fracture and limited periosteal reaction on the right tibia at the lateral condyle level (epiphyseal-metaphyseal area). No mineralized matrix of the lesion was seen. The tibial surface of the knee joint showed uneven irregularities on the lateral portion adjacent to the condylar lesion (Figure 3D). These changes are compatible with a benign process, likely a giant cell tumor (GCT). Additionally, subchondral sclerosis was observed at the remaining tibial condyles, possibly due to osteoarthritis.

Individual #4 exhibited bone islands on the right iliac crest, right femoral neck, and in the sacral 2nd and 3rd vertebrae. Finally, individual #8 showed a lesion on the right iliac crest revealing the “polka dot” sign typical of intraosseous hemangioma, another benign neoplasm (Figure 3E). The same individual also displayed bone islands within the body of the 3rd lumbar vertebra (Figure 3F) (24).

### Lung calcification

**Figure 4:**
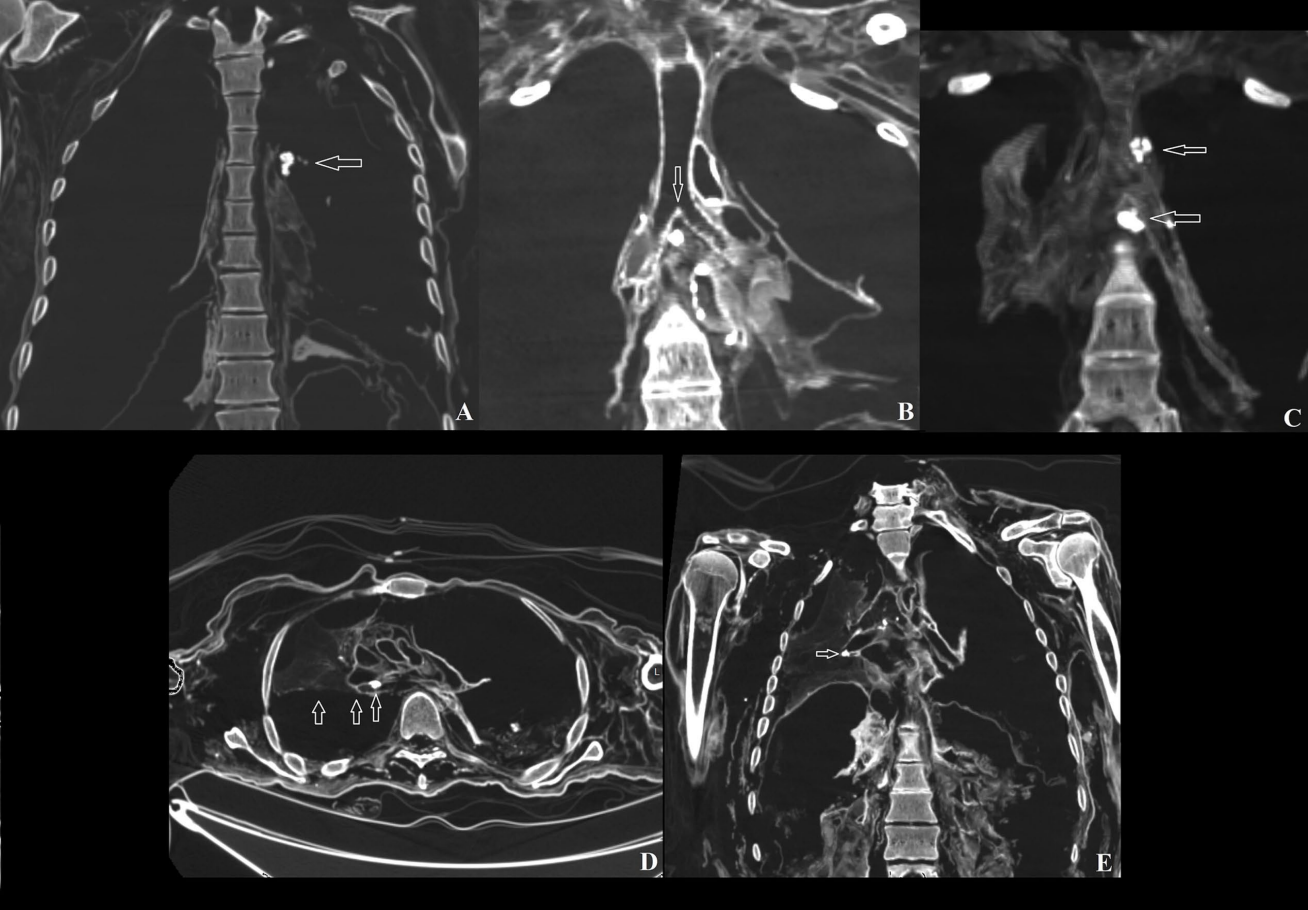
**(A)** Anterior lung calcification in individual #1; **(B)** Lymph node calcification in individual #8; **(C)** Carinal lymph node and left pleural lymph node calcifications in individual #14; **(D)** Mediastinal shift in individual #15; **(E)** Right pleural adhesions in individual #15.

Calcareous deposits are frequently observed in the lungs due to various factors (25). Individual #1 exhibited a calcification (pleural or calcified lymph node) measuring 1.4 × 0.6 cm, along with a 0.4 cm calcification in the anterior part of the thoracic cavity (Figure 4A), and multiple left superior-posterior calcifications within the thoracic cavity. Individual #8 showed calcification in the left collapsed lung and below the bronchial carina (Figure 4B). A sub-carinal calcification and adjacent pleural calcifications were observed in individual #14, while calcifications at the sub-carinal, right hilar, and interlobar lymph nodes, as well as pleural adhesions with a suspected right upward mediastinal shift, were seen in individual #15 (Figures 4C–E).

### Cardiovascular calcification

**Figure 5:**
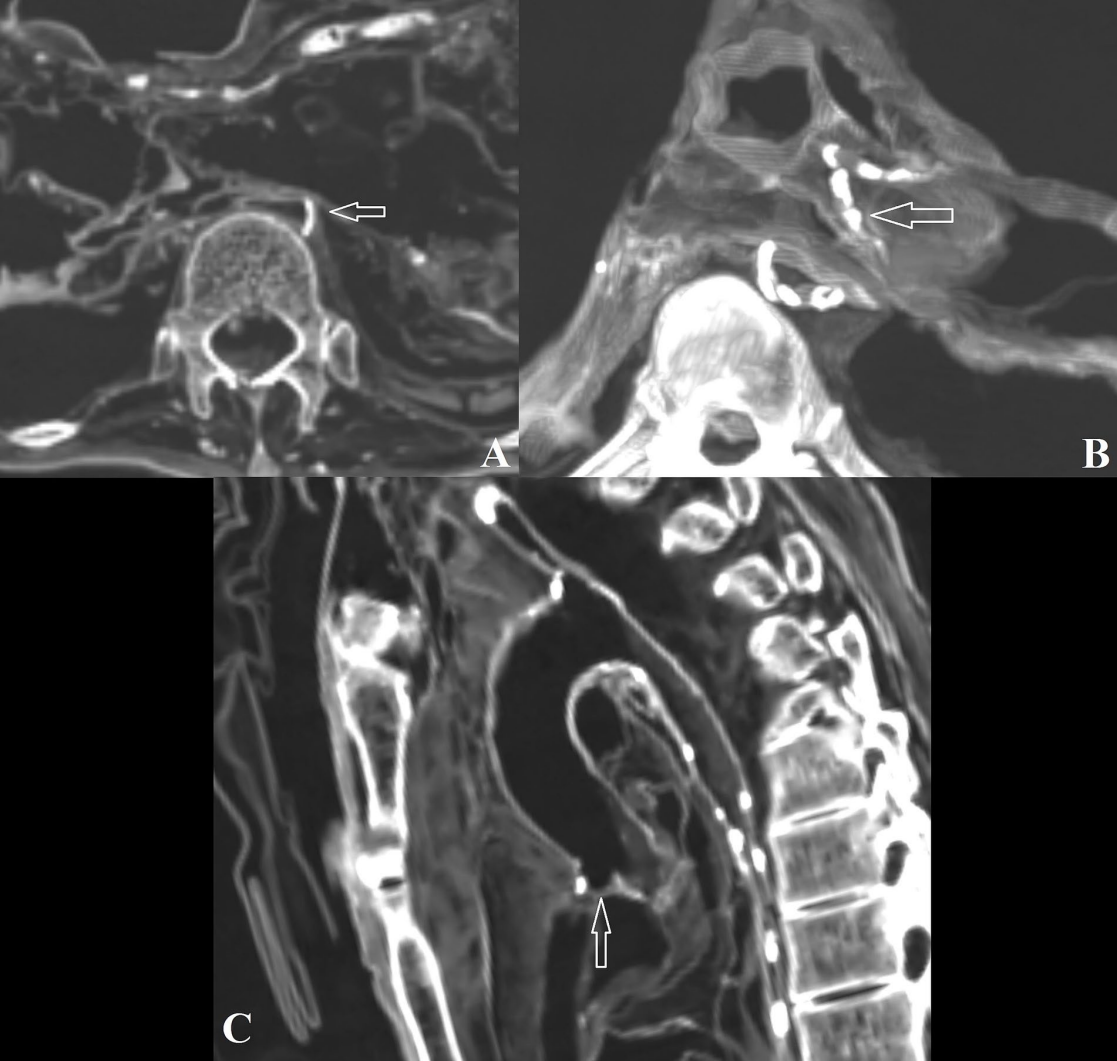
**(A)** Aortic calcification in individual #4; **(B)** Left coronary artery calcification in individual #8; **(C)** Aortic valve calcification in individual #8.

Cardiovascular calcification is the process by which mineral deposits form in the vessels and heart (26). Individual #4 exhibited aortic calcification, while individual #8 revealed calcification in the aorta, aortic valve, and coronary arteries, with the abdominal segment heavily calcified (Figures 5A–C).

### Miscellaneous conditions

**Figure 6:**
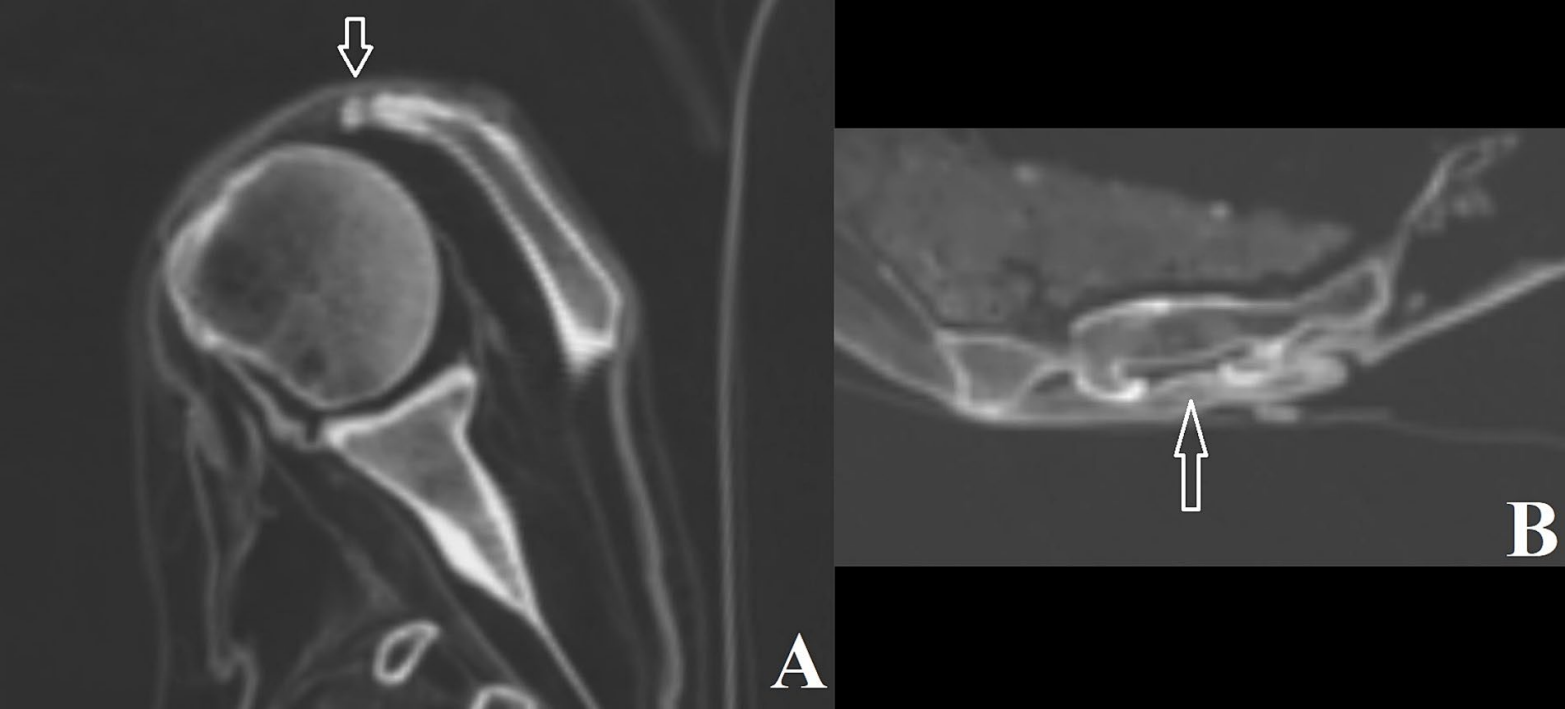
**(A)** *Os acromiale* in individual #0; **(B)** Closed sacral vertebral dysraphism at vertebra 4 in individual #1.

Miscellaneous conditions and anatomical variations were also identified in this study (13, 27). Individual #0 exhibited a calcaneal spur on the right side and a left pre-acromion *os acromiale* variation (Figure 6A). Individual #1 showed a left persistent ulnar styloid ossicle (with a fracture being less likely) and a neural tube defect in sacral vertebra 4, typical of closed spinal dysraphism (Figure 6B). Additionally, individual #4 exhibited this condition, affecting S4 and S5.

### Pseudopathology and post-depositional alterations

**Figure 7:**
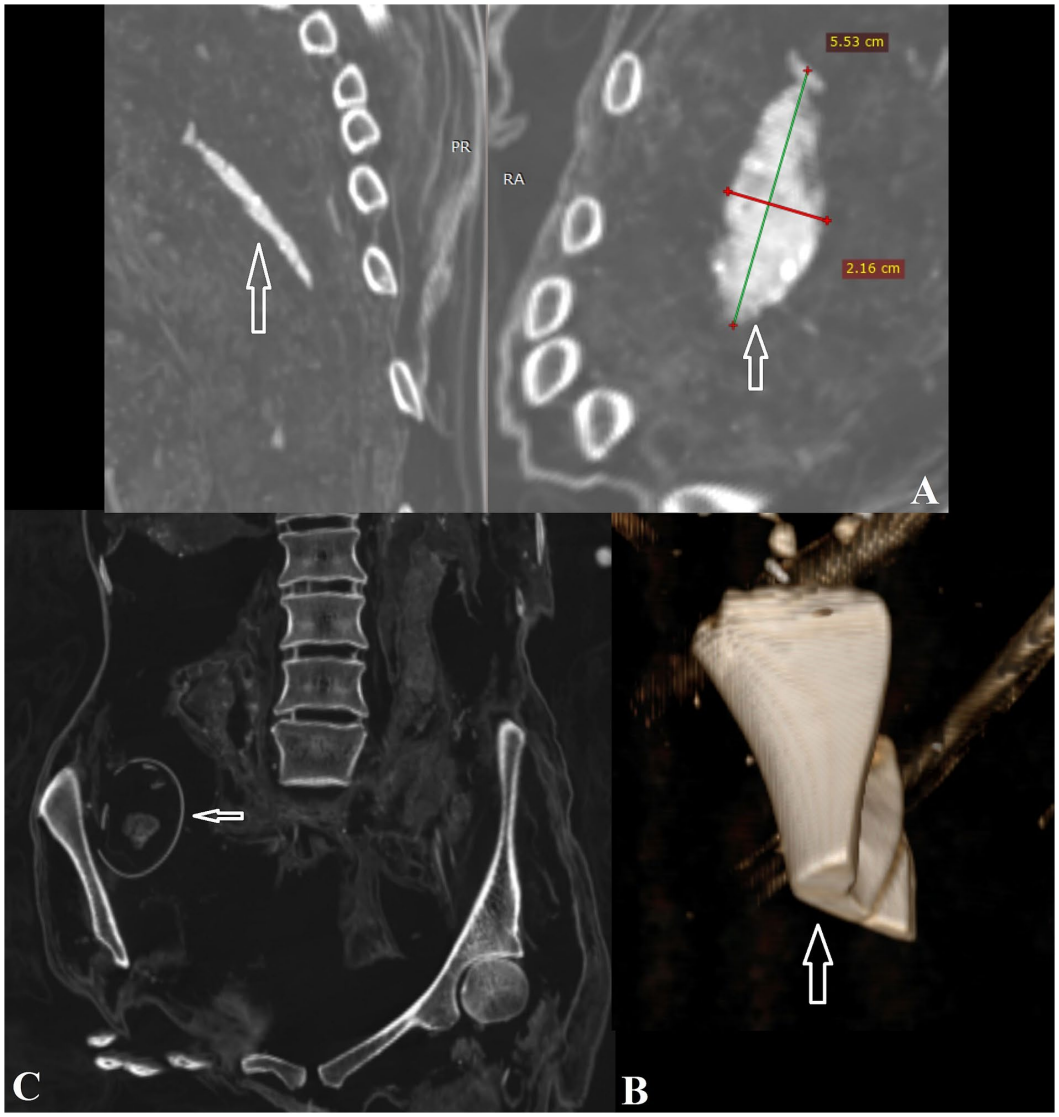
**(A)** Unidentified object in the right thoracic cavity of individual #0; **(B)** Butchered animal bone in the thoracic cavity of individual #3; **(C)** Eggshell inside the pelvic cavity of individual #3.

Pseudopathology refers to cases where features observed on remains may mimic a pathological condition, but are actually due to other post-depositional or taphonomic factors, such as animal activity or manipulation (28). Individual #0 contained an unidentified object measuring 5.5 × 2.1 cm inside the right thoracic cavity (Figure 7A). Individual #3 had an egg measuring 5.9 × 4.3 cm in the pelvic cavity on the right side. A fragment of butchered animal bone was found inside the abdominal cavity on the right side (Figures 7B,C), while another fragment was detected in the thoracic cavity. In individual #4, bones, possibly tarsals, were observed inside the chest. This individual also had a metal object (possibly a nail) inside the thoracic cavity and another beneath the wrist.

## Discussion

The mummified remains central to this article provide a valuable source of information, both historically and biomedically (1). Given the significance of the Radziwiłł family and their role in Lithuanian history, periodic evaluations of the remains were necessary to monitor their condition. Differential preservation of some of the remains suggested that, since the last analysis in 1987, several individuals lost their soft tissue and became skeletonized due to environmental changes over time. This indicates that the conservation conditions within the chambers were not optimal for the long-term preservation of these remains, justifying the museum’s decision to produce new coffins and restructure the area. As mentioned earlier, we confirmed that some of the skulls were missing from the bodies, likely due to events during the Soviet occupation of Lithuania, when religious practices were suppressed, and churches were often repurposed for other uses (4).

Concerning the mummification process, the majority of the remains we examined were spontaneously mummified, likely due to the low temperatures in the crypts, protection from rainwater by the overlying church structure, and burial in wooden coffins that formed a barrier against the surrounding environment (1). Burial in subterranean chambers is a widespread practice across Europe, often linked to individuals of high social status as is the case for the Kėdainiai community (3). Only one case involved artificial mummification, specifically the body identified as Janusz II Radziwiłł (1612-1655) (individual #0). He was embalmed and placed in a metal coffin (sarcophagus #2), and is the only individual for whom the identity could be somewhat confirmed. Inspection of his body revealed a capsized U-shaped, suture-closed abdominal incision, with the cavity stuffed with common hops (*Humulus lupulus*), known for its antimicrobial properties (29). A similar process of evisceration and stuffing was documented starting in the Middle Ages for prominent individuals and aristocrats, such as the Aragonese in Naples and the Capaccis in Siena, Italy (30, 31). This special treatment may have been due to the transportation of his body from Tykocin (Poland) to the crypt, further indicating his elevated status. Assuming a mechanical origin, his bipartite acromion—previously observed in another mummy from Vilnius (32)—may suggest chronic tearing of the rotator cuff due to stress, while the presence of Schmorl’s nodes indicates flexion and bending of the vertebral column (27, 33, 34). Additionally, pseudopathology and post-mortem alterations, such as the eggshell in the pelvic cavity and two fragments of animal bone in one of the mummies, suggest animal (possibly marten) activity and minimal care for the remains over time (35).

Turning to pathological conditions, evidence of age- or activity-related degenerative changes in the joints—such as altered articular contours, lipping, osteophyte formation, and cyst development—was found in five individuals. One case showed signs of sacroiliitis, possibly related to non-specific spondyloarthropathy (13). Although degenerative joint disease is a multifactorial condition, it is often regarded as age-related, resulting from the continuous mechanical loading of joints or the spine throughout an individual’s life (36). Findings from a roughly coeval sample of skeletal remains from Vilnius indicate that age was a significant factor in the prevalence of degenerative disease in both males and females, likely also applying to the majority of the mummies examined, who survived into adulthood (37).

Calcifications were observed in the chest cavities of four individuals, indicating lung disease among the Polish-Lithuanian upper classes (38). These findings are comparable to those in other coeval mummy contexts, such as a male from Sommersdorf, Germany, and a female from Leuk, Switzerland (39, 40). In paleopathology, calcified pulmonary nodules and fibrotic changes are commonly associated with tuberculosis (1, 41, 42). Ancient DNA testing has confirmed that these calcifications are related to tubercle bacillus infections, as seen in the Vác mummies from Hungary (43, 44). This disease was endemic during the relevant time frame, narrowing down the range of possible conditions affecting the Kėdainiai individuals. However, other pathological processes with similar lung lesions, such as histoplasmosis, coccidioidomycosis, pneumoconiosis, and sarcoidosis, though less likely, should also be considered (25).

A calcified abdominal aorta was found in two cases, one of which also showed coronary and aortic valve calcifications (26). Although extensive tissue sampling for histological confirmation was not permitted, the most likely explanation for these findings, as emphasized in a similar study of a 17th-century Korean mummy from Mungyeong, is atherosclerosis (45). This is not surprising, considering the highly atherogenic diets of Lithuanian elites, a fact also supported by historical records (32, 46). However, other conditions such as age, diabetes, metabolic disorders, localized inflammation, and kidney disease could also result in similar calcifications, with filariasis and hyperparathyroidism ruled out (47).

This study also identified benign tumors in the sample (48). The vertebral hemangioma and intraosseous hemangioma observed are of particular paleopathological interest, as they have been rarely documented in the literature. A case affecting the skull was identified in a skeleton from the Chinese archaeological site of Redianchang (49), and another was found in a Roman-period skeleton from the Dakhleh Oasis in Egypt (50). Differentials include Paget’s disease, which may sometimes be confused with hemangioma. The other tumor case found in this study is also noteworthy. Its location on the epiphyseal-metaphyseal area of the proximal tibia, along with its solitary, eccentric, and osteolytic appearance with smooth edges, suggests a giant cell tumor (GCT)—a benign tumor that can occasionally become aggressive. Other conditions such as aneurysmal bone cyst, chondroblastoma, and non-ossifying fibroma are less likely due to the adult age of the individual, and cancer was ruled out due to the absence of bone reaction and the regular appearance of the lesion contours. The lesion also showed a cortical break in the lateral border, which could have led to a limited periosteal reaction, while the sclerotic and irregular condylar surface might indicate a micro-pathological fracture. A similar case was recently discovered in an 18th-century Italian mummy from Roccapelago (51). However, it is important to note that some conditions may mimic GCT, and without clinical data, the diagnosis must remain tentative (52). Additionally, bone islands, which are benign, non-cancerous bone lesions, were also identified. These often-overlooked findings contribute to the understanding of non-cancerous skeletal formations in ancient populations (13, 53). Malignant neoplasms can result from various oncogenic factors, such as mutations, viruses, bacteria, or even specific dietary habits, such as high meat consumption among European upper classes (46, 54). In this assemblage, these tumors likely went unnoticed due to the inability to conduct dissections and histological evaluations of internal organs (55).

## Conclusion

In conclusion, the CT investigation of eight high-status members of the Kėdainiai historic community allowed the assessment of incidental pathological conditions in a set of mummified remains, which could partly be explained by environmental influences. These findings provide a narrative of social status, disease, and post-mortem manipulation. Although providing a definitive diagnosis for some of the conditions was challenging due to the lack of clinical history, as well as potential alterations to the inner organs caused by taphonomic factors (56), the imaging study lays the foundation for future investigations using biopsy and other biomedical techniques. These techniques can maintain the integrity of the bodies while avoiding major damage (57), and further advocate for periodic monitoring of the remains, ensuring their continued potential for storytelling through future research and conservation efforts (58).

Beyond the specific diagnoses, this study also reveals the broader historical, cultural, and biological context of the population, shedding light on how the individuals faced challenges such as bone degeneration, respiratory infections, and cardiovascular issues, as well as other non-cancerous conditions rarely represented in the bioarchaeological literature. It also offers insights into their funerary rituals and the impact of political changes that led to the profanation of their graves. This broader context not only helps address key research questions about their social status, health, and cultural practices, but also frames their lives within 17th- and 18th-century Lithuania (59, 60). Paleopathology, coupled with paleoradiology, remains an anatomical discipline devoted to exploring disease in once-living societies through non-destructive or minimally invasive methods, maintaining the dignity of human remains while providing the most realistic explanations for the lesions observed.

## Data Availability

The original contributions presented in the study are included in the article/supplementary material, further inquiries can be directed to the corresponding author.
